# A Novel and Practical Screening Tool for the Detection of Silent Myocardial Infarction in Patients With Type 2 Diabetes

**DOI:** 10.1210/jc.2016-1318

**Published:** 2016-06-14

**Authors:** Peter P. Swoboda, Adam K. McDiarmid, Bara Erhayiem, Philip Haaf, Ananth Kidambi, Graham J. Fent, Laura E. Dobson, Tarique A. Musa, Pankaj Garg, Graham R. Law, Mark T. Kearney, Julian H. Barth, Ramzi Ajjan, John P. Greenwood, Sven Plein

**Affiliations:** Multidisciplinary Cardiovascular Research Centre & Leeds Institute of Cardiovascular and Metabolic Medicine (P.P.S., A.K.M., B.E., P.H., A.K., G.J.F., L.E.D., T.A.M., P.G., M.T.K., R.A., J.P.G., S.P.), University of Leeds, Leeds, United Kingdom; Leeds Teaching Hospitals NHS Trust (A.K., J.H.B.), Leeds, United Kingdom; Division of Epidemiology and Biostatistics (G.R.L.), Leeds Institute of Cardiovascular and Metabolic Medicine, University of Leeds, Leeds, United Kingdom

## Abstract

**Objective:**

Silent myocardial infarction (MI) is a prevalent finding in patients with type 2 diabetes and is associated with significant mortality and morbidity. Late gadolinium enhancement (LGE) by cardiovascular magnetic resonance (CMR) is the most validated technique for detection of silent MI, but is time-consuming, costly, and requires administration of intravenous contrast. We therefore planned to develop a simple and low-cost population screening tool to identify those at highest risk of silent MI validated against the CMR reference standard.

**Methods:**

A total of 100 asymptomatic patients with type 2 diabetes underwent electrocardiogram (ECG), echocardiography, biomarker assessment, and CMR at 3.0T including assessment of left ventricular ejection fraction and LGE. Global longitudinal strain from two- and four-chamber cines was measured using feature tracking.

**Results:**

A total of 17/100 patients with no history of cardiovascular disease had silent MI defined by LGE in an infarct pattern on CMR. Only four patients with silent MI had Q waves on ECG. Patients with silent MI were older (65 vs 60, *P* = .05), had lower E/A ratio (0.75 vs 0.89, *P* = .004), lower GLS (–15.2% vs –17.7%, *P* = .004), and higher amino-terminal pro brain natriuretic peptide (106 ng/L vs 52 ng/L, *P* = .003). A combined risk score derived from these four factors had an area under the receiver operating characteristic curve of 0.823 (0.734–0.892), *P* < .0001. A score of more than 3/5 had 82% sensitivity and 72% specificity for silent MI.

**Conclusions:**

Using measures that can be derived in an outpatient clinic setting, we have developed a novel screening tool for the detection of silent MI in type 2 diabetes. The screening tool had significantly superior diagnostic accuracy than current ECG criteria for the detection of silent MI in asymptomatic patients.

## Teaser

The rate of silent MI in this cohort of patients with type 2 diabetes was 17%. A screening tool for its detection was created from clinical parameters including age, E/A ratio, GLS and NT-proBNP.

Cardiovascular disease, primarily stroke and myocardial infarction (MI), account for the vast majority of mortality associated with type 2 diabetes (1, 2). Silent MI is a relatively common finding in patients with type 2 diabetes (3, 4), although the exact prevalence in contemporary asymptomatic populations is unknown (5).

Currently, the most extensively validated method to assess for the presence and extent of silent MI is the late gadolinium enhancement (LGE) technique measured by cardiovascular magnetic resonance (CMR). Using this technique, it is possible to establish the location and distribution of scar tissue. The prevalence of silent MI according to the presence of LGE in symptomatic patients with type 2 diabetes is reported to be between 21 and 28% (3, 4). In these cohorts, the presence of silent MI was strongly associated with an increase in major adverse cardiovascular events and mortality.

There has been a decrease in the rate of acute MI in patients with type 2 diabetes over the past two decades (6). This reduction is thought to reflect improvements in glycemic control and modification of other concomitant risk factors such as smoking, dyslipidemia, and blood pressure (BP). However, per definition, silent MI is usually undetected and affected patients therefore fail to benefit from aggressive risk factor management, which may explain the poor clinical outcome in this group.

The detection of patients with silent MI remains a challenge as the most accurate method relies on CMR, which has limited availability, is relatively time consuming, costly and requires administration of IV contrast, making it a less than ideal population screening tool. Several imaging and biomarker tests have been shown to be able to detect the presence and determine the extent of clinically recognized MI measured by LGE including Q waves on a 12-lead electrocardiogram (ECG) (7), ejection fraction (8), strain parameters (9), high sensitivity troponin (hs-cTnT) (10), and amino-terminal probrain natriuretic peptide (NT-proBNP) (11). However, the sensitivity and specificity of these tests to detect silent MI in type 2 diabetes is at present unknown.

In this study, we aimed to assess the diagnostic accuracy of several commonly measured parameters in asymptomatic patients with type 2 diabetes to detect silent MI. We hypothesized that a risk score derived from a combination of these measurements could accurately predict the presence of silent MI on CMR.

## Materials and Methods

### Enrollment criteria

A total of 100 asymptomatic patients with type 2 diabetes were recruited from 30 primary care health centers in West Yorkshire, UK. The study was approved by the local ethical committee (13/YH/0098) and individuals were enrolled onto the study after informed consent. Exclusion criteria were known cardiovascular disease (including ischemic heart disease, heart failure or persistent atrial fibrillation), kidney disease (estimated glomerular filtration rate < 30), uncontrolled hypertension (with latest BP >140/80 mm Hg (12)), treatment with insulin or angiotensin-converting enzyme inhibitor/angiotensin receptor blocker (to avoid patients with occult evidence of renal or other end-organ damage).

### CMR protocol

All patients underwent an identical CMR study on a dedicated cardiovascular 3 Tesla Philips Achieva TX system (Philips) equipped with a 32-channel coil and MultiTransmit technology. Data were acquired during breath-holds at end-expiration.

From scout CMR images, the left ventricular (LV) long and short axes were determined. Cine images covering the entire heart in the LV short axis plane and orthogonal long-axis planes were then acquired (balanced steady-state free precession, spatial resolution 1.2 × 1.2 × 10 mm^3^, 50 cardiac phases repetition time/echo time 2.6/1.3 ms, flip angle 40°, field of view 300–420 mm). Cines planned to cover the entire left atrium short axis plane in end-systole were also acquired (as in LV stack but with a slice thickness of 5 mm).

Late gadolinium enhancement (LGE) imaging was carried out more than 6 minutes after contrast injection (0.15 mmol/kg Gadovist, Bayer Schering) using inversion recovery-prepared T1-weighted echo. The optimal inversion time to null signal from normal myocardium was determined using a Look-Locker approach. Typical parameters are repetition time/echo time 3.5/2.0 ms, flip angle 25°, acquired spatial resolution 1.54 × 1.76 × 10 mm^3^ and performed in 10–12 short axis slices with further slices acquired in the vertical and horizontal long axis orientations, phase-swapped or imaged in systole, if indicated based on LGE imaging obtained or wall-motion abnormality.

### CMR interpretation

CMR data were assessed quantitatively using commercially available software (CVI42 v5.1.0, Circle Cardiovascular Imaging Inc.) blinded to clinical details. LV mass, ejection fraction, and left atrial volume were measured from short axis cine images.

For feature tracking analysis, endocardial and epicardial LV contours were drawn on long axis four-chamber and two-chamber cines using a semiautomated process. Peak global longitudinal strain, systolic strain rate, and early and late diastolic strain rates were measured. Late diastolic strain rates were defined as peak rate during active atrial contraction.

The presence of silent MI was identified by two physicians experienced (5 and 15 years) in CMR interpretation based upon typical subendocardial distribution of LGE present. The mass of LGE was quantified by the Otsu method (13).

### Echocardiography, ECG, and 24-hour blood pressure monitoring

All patients underwent echocardiography (Vivid e9, GE Medical Systems) focused on Doppler measurements of mitral inflow and tissue Doppler imaging of the lateral and medial mitral annulus. E/A ratio (the inverse was used for the index), E′, A′, and S′ are measured on the machine using inbuilt software. Diastolic dysfunction was graded 0–3 by an accredited echocardiographer blinded to clinical details according to international guidelines (14). Twelve-lead electrocardiography (MAC500, GE Medical Systems) was analyzed by two physicians blinded to clinical details for the presence of Q waves according to international guidelines (15). All patients underwent 24 hour BP monitoring (6100, Welch-Allyn) set to inflate every 30 minutes in the day and every hour at night.

### Blood tests

Blood was drawn from each subject at the time of CMR and tested for HbA1c. Serum was stored at –70 C and tested in one batch for hs-cTnT typical coefficient of variability 4.4% at 13.7 ng/liter and 3.6% at 95.3 ng/liter, NT-proBNP typical coefficient of variability 2.9% at 91 ng/liter and 2.1% at 415 ng/liter (Cobas 8000, Roche Diagnostics, Burgess Hill, West Sussex) and hs-CRP (Advia, Siemens Healthcare Diagnostics, Marburg, Germany). Fasting cholesterol values were recorded from review of electronic records.

### Statistical analysis

Statistical analysis was performed using IBM SPSS Statistics 22.0 (IBM Corp.). Continuous variables were expressed as means ± standard deviation and compared using *t* test or Mann Whitney U test depending on normality. Categorical variables were expressed as N (%) and compared using Fisher exact test.

Receiver operating characteristic (ROC) analysis was used to determine the diagnostic accuracy of age, E/A ratio, GLS, and NT-proBNP for the detection of silent MI. These parameters were chosen because they were significantly different in those with silent MI and have biological validity. The diagnostic accuracy is expressed as area under the curve (AUC) and 95% confidence interval (CI). Optimal sensitivity and specificity were calculated using Youden index. Nested models were used to establish the best possible AUC from combining the variables associated with silent MI. AUCs were compared by using validated methods described by DeLong et al (16).

Using the cutoffs derived from the Youden analysis of the ROC curves, each variable was given a binary classification (0 or 1). These four categorical variables were summed to calculate a silent MI risk score (range 0–4). The AUC of the silent MI risk score was compared to the best possible AUC from the individual parameters derived from the nested risk model.

We estimated that for a screening tool to be clinically useful it would need sensitivity a of more than 80% and to be significantly superior to the current screening test of Q waves, which has a sensitivity of 28% (7). Assuming that the prevalence of silent MI in a diabetic cohort to be around 10% (4) using a two-sided Fisher exact test with α = 0.05 and power 80%, it was calculated 98 patients would be needed, including 10 with silent MI. *P* < .05 two-sided was considered statistically significant.

## Results

**Figure 1.**
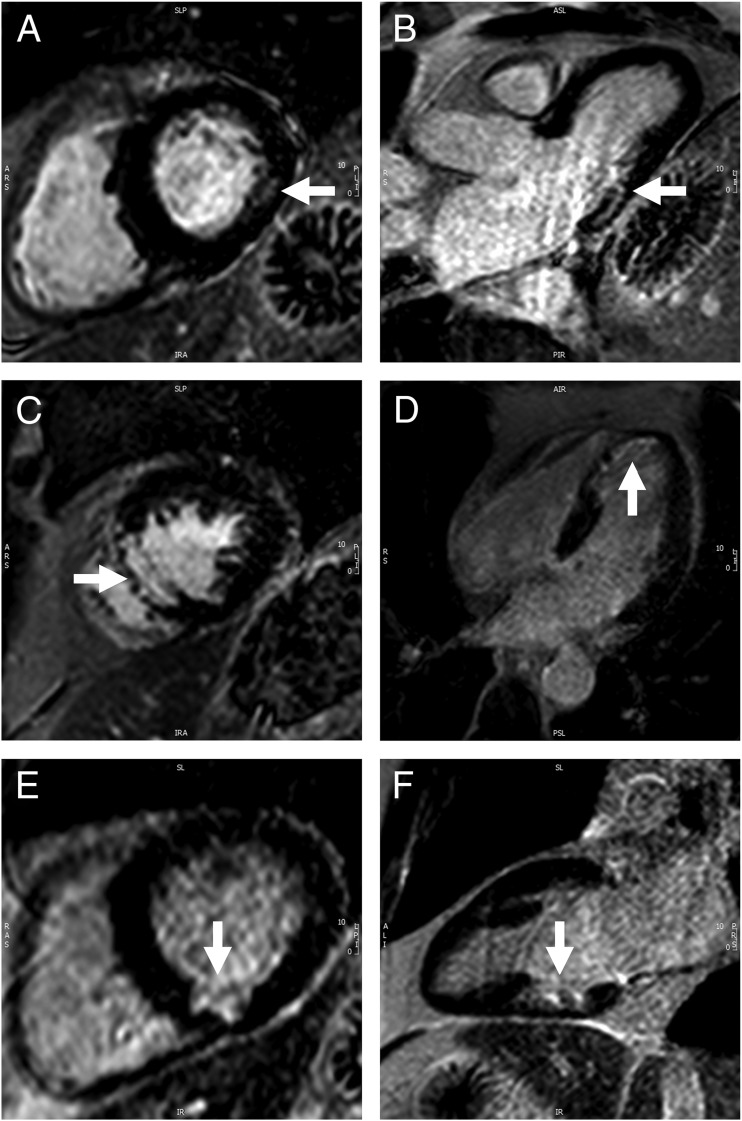
Examples of silent MI detected by LGE. Horizontal panels are from the same patient and white arrows denote the area of MI. (A, B) Basal and mid-inferolateral subendocardial MI. (C, D) Apical and mid septal near transmural infarction. (E, F) Basal inferior subendocardial infarction.

Seventeen of the 100 patients had evidence of silent MI defined as a subendocardial pattern of LGE identified by two experienced CMR reporters independently. Figure 1 shows examples from three patients. For the whole population, mean ± SD age was 60.7 ± 10.9 years, duration of diabetes 5.0 ± 4.4 years, current HbA1c 63.1 ± 19.6 mmol/mol, median HbA1c since diagnosis 64.5 ± 17.2 mmol/mol, and 24-hour BP 131.4 ± 15.0/72.7 ± 9.1 mm Hg. Of the 100 patients, 82 were male, 72 were white British, 19 South Asian, 6 Black, 1 Turkish, 1 Polish, and 1 Latin American. Patient characteristics are shown in Table 1 according to silent MI status. There was a range in the extent of silent MI from 0.4 g to 36.6 g. Mean mass of infarction was 6.1 ± 8.8 g (5.8 ± 8.5% of LV mass) and was predominantly subendocardial with mean transmurality of 60.3 ± 28.0%.

**Table 1.** Patient Characteristics and Investigation Findings According to the Presence or Absence of Silent MI

	Silent MI	No Silent MI	*P* Value
N	17	83	
Age, y	65.4 ± 9.2	59.8 ± 11.0	**.05**
Male gender, n (%)	16 (94)	66 (80)	.30
Body mass index, kg/m^2^	27.8 ± 3.1	28.9 ± 4.6	.32
Duration of diabetes, y	4.1 ± 4.1	5.2 ± 4.4	.24
Current HbA1c, mmol/mol	57.1 ± 12.5	64.3 ± 20.6	.23
Median HbA1c since diagnosis, mmol/mol	63.3 ± 10.9	64.8 ± 18.2	.77
24-h systolic BP, mm Hg	135.3 ± 15.9	130.8 ± 14.7	.24
24-h diastolic BP, mm Hg	72.5 ± 10.1	72.7 ± 8.9	.89
Total cholesterol	4.3 ± 1.2	4.4 ± 1.1	.69
Smoking, n (%)	4 (24)	11 (13)	.28
Ethnicity			.84
White British	12 (71)	60 (72)	
South Asian	4 (24)	15 (18)	
Black	1 (6)	5 (6)	
Other^a^	0 (0)	3 (4)	
Metformin, n (%)	13 (76)	74 (89)	.23
Sulphonylurea, n (%)	5 (29)	28 (38)	1.0
Gliptin, n (%)	2 (12)	9 (11)	1.0
Exenatide, n (%)	0	1 (1)	1.0
Glitazone, n (%)	0	1 (1)	1.0
Repaglinide, n (%)	0	1 (1)	1.0
Dapagliflozin, n (%)	0	1 (1)	1.0
Insulin, n (%)	0	0	—
ACE inhibitor, n (%)	0	0	—
*β* blocker, n (%)	2 (12)	2 (2)	.13
Calcium channel blocker, n (%)	4 (24)	6 (7)	.06
Diuretic, n (%)	1 (6)	4 (5)	1.0
Statin, n (%)	14 (82)	56 (68)	.26
Fibrate, n (%)	0	0	—
Ezetimibe, n (%)	0	0	—
Aspirin, n (%)	2 (12)	16 (20)	.73
CMR			
LVEDV, ml	140.5 ± 39.1	150.0 ± 32.8	.30
LVEDV index, ml/m^2^	70.4 ± 17.1	74.5 ± 13.4	.27
Ejection fraction, %	58.0 ± 9.7	61.7 ± 4.9	.30
LV mass, g	102.5 ± 16.3	95.0 ± 21.0	.34
LV mass index, g/m^2^	51.4 ± 6.5	47.2 ± 8.7	**.01**
LA volumes, ml	89.0 ± 31.6	88.5 ± 16.8	.93
LA volume index, ml/m^2^	44.9 ± 15.9	44.2 ± 7.7	.87
Feature tracking			
GLS	−15.2 ± 3.7	−17.7 ± 3.1	**.004**
SSR	−93.8 ± 31.8	−111.2 ± 42	**.04**
EDSR	64.1 ± 16.6	84.0 ± 33.1	**.02**
LDSR	87.4 ± 39.9	91.4 ± 41.2	.89
Echocardiography			
E/A ratio	0.75 ± 0.30	0.89 ± 0.30	**.03**
E/E′ average	7.4 ± 2.4	7.1 ± 2.1	.96
S′ average, cm/s	9.8 ± 2.2	9.5 ± 1.8	.72
Electrocardiography			
Q waves (%)	4 (24)	6 (7)	.06
Biomarker findings			
hs-cTnT, ng/liter	7.5 ± 4.1	7.4 ± 5.4	.42
NT-proBNP, ng/liter	105.8 ± 132.2	51.9 ± 100.8	**.003**
hs-CRP, mg/liter	3.5 ± 3.5	3.7 ± 5.9	.57

Abbreviations: ACE, angiotensin-converting enzyme; EDSR, early diastolic strain rate; hs-CRP, high-sensitivity C-reactive protein; LA, left atrial; LDSR, late diastolic strain rate; LVEDV, left ventricular end-diastolic volume; SSR, systolic strain rate. ^a^Other ethnicities; 1 Turkish, 1 Polish, and 1 Latin American.

Patients with silent MI were older than those without silent MI (65.4 ± 9.2 vs 59.8 ± 11.0 years, *P* = .05), but there was no significant difference in any other patient characteristic or use of medication. There was no significant difference in any cardiac risk factors including 24-hour BP, fasting cholesterol, duration of diabetes, or smoking (*P* = .24, 0.69, .24, and .28, respectively).

The mean number of previous HbA1c measurements included in the analysis was 9.7 ± 5.7 per patient over 4.3 ± 2.7 years. There was no significant difference between mean, median, or highest HbA1c since diagnosis between those with and without silent MI (*P* = .69, .77, and .28. respectively).

### ECG

Pathological Q waves on ECG were only present in 4/17 with silent MI and 6/83 without silent MI (sensitivity 24%, specificity 93%). Other ECG abnormalities present in 19/100 patients were not associated with silent MI and included left axis deviation in five, right bundle branch block in five, LV hypertrophy by voltage criteria in four, left anterior hemiblock in three, T wave abnormalities in three, and trifascicular block in one.

### Echocardiography

Results of echocardiography are shown in Table 1. The only significant difference between those with and without silent MI was a lower E/A ratio (0.75 ± 0.30 vs 0.89 ± 0.30, *P* = .03) in patients with silent MI. Grade of diastolic dysfunction was not significantly different between those with and without silent MI (grade 0, 6 vs 19%; grade 1, 88 vs 75%; grade 2, 0 vs 5%; and grade 3, 6 vs 1% *P* = .24).

### CMR

CMR results are shown in Table 1. LV mass indexed to body surface area was higher in those with silent MI than those without (51.4 ± 6.5 vs 47.2 ± 8.7 g/m^2^, *P* = .01). There was no other difference in volumetric parameters. Of the longitudinal strain parameters measured by feature tracking, GLS –15.2 ± 3.7 vs –17.7 ± 3.1%, *P* = .004, peak systolic strain rate –93.8 ± 31.8 vs –111.2 ± 42%, *P* = .04, and early diastolic strain rate 64.1 ± 16.6 vs 84.0 ± 33.1%, *P* = .02, were all significantly lower in those with silent MI. There was no difference in late diastolic strain rate, *P* = .89.

Of the patient characteristics shown in Table 1, none had a significant association with quantitative mass of silent MI. Of the investigation findings shown in Table 1 the mass of silent MI only had significant correlations with LV ejection fraction (R = –0.81, *P* < .0001), E/E′ (R = –0.58, *P* = .02), and hs-cTnT (R = 0.58, *P* = .02).

### Biomarkers

NT-proBNP was significantly higher in those with silent MI (105.8 ± 132.2 vs 51.9 ± 100.8 ng/liter, *P* = .003). There was no difference in hs-CRP or hs-cTnT (*P* = .57 and 0.42, respectively).

### Development of a screening tool

The area under the ROC curve for age, Q waves, E/A ratio, GLS, and NT-proBNP are shown in Table 2 and the Supplemental Figure 1. The AUC for the nested model of all four variables was 0.850 (0.765–0.914), *P* < .0001, and the maximum possible sensitivity was 94% and specificity 71%. The nested model had higher diagnostic accuracy than Q waves, age, E/A ratio, and GLS alone (*P* <.0001, .02, .02, and .006, respectively). The improvement over NT-proBNP showed a trend (*P* = .07). The addition of Q waves did not significantly improve the AUC of the model.

**Table 2.** AUC of Q Waves and the 4 continuous Parameters for Detecting Silent MI

	AUC	*P* Value	Optimum Cutoff	Sensitivity at Cutoff (%)	Specificity at Cutoff (%)
Q waves	0.582 (0.421–0.742)	.29	Categorical	24	93
Age	0.668 (0.522–0.803)	.02	>62	76	63
E/A ratio	0.669 (0.526–0.813)	.02	≤0.79	71	59
GLS	0.685 (0.542–0.829)	.01	≥−18.4%	88	41
NT-proBNP	0.730 (0.604–0.855)	<.001	>29 ng/liter	88	57

Optimum cutoff, sensitivity, and specificity derived from Youden index are also shown.

**Figure 2.**
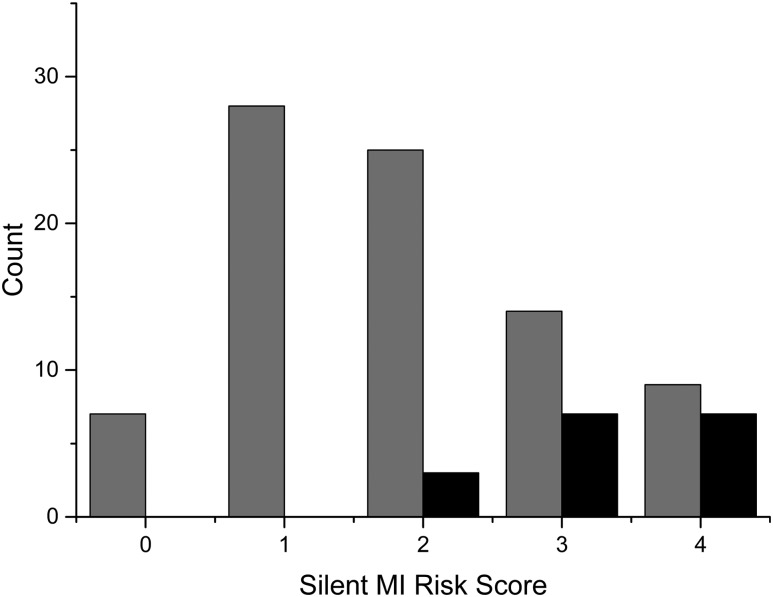
Number of patients with silent MI (black) and without silent MI (gray) according to their silent MI risk score.

The combined four-variable silent MI risk score had an AUC of 0.823 (0.734–0.892), *P* < .0001, and better diagnostic accuracy than Q waves, age, or E/A ratio alone (*P* < .0001, .001, and .02, respectively). The sensitivities and specificities for each possible silent MI risk score are shown in Table 3. The number of patients with silent MI according to their silent MI risk score is shown in Figure 2.

**Table 3.** Silent MI Risk Score Calculated From Age >62, GLS ≥ −18.4%, EA Ratio ≤ 0.79, and NT-proBNP > 29 ng/liter

Silent MI Risk Score	Sensitivity	95% CI	Specificity	95% CI
0	100.0		0.0	
≥1	100.0		0.0	
≥2	100.0	80.5–100.0	42.2	31.4–53.5
≥3	82.4	56.6–96.2	72.3	61.4–81.6
≥4	41.2	18.4–67.1	89.2	80.4–94.9

The sensitivity and specificity to detect silent MI for each possible score is shown.

## Discussion

The prevalence of silent MI (17%) detected by LGE imaging in this low-risk asymptomatic population was high approaching one in five patients. We have found increasing age to be the only conventional risk factor associated with silent MI. We have identified several markers of silent MI that can be detected by cardiac imaging or blood test and have shown that these markers can be combined to develop a simple screening tool with good diagnostic accuracy.

We have demonstrated that a simple risk score can predict the presence of silent MI in patients with type 2 DM as shown by LGE on CMR. The risk score is composed of age, E/A ratio below 0.79, GLS greater than –18.4%, and NT-proBNP above 29 ng/liter. These are all parameters that are often measured in a cardiology clinic or could easily be measured in a community-based screening. In the model, we derived GLS from feature tracking of CMR cine images; however, it is possible to measure GLS from standard echocardiography, which has been demonstrated to show good agreement with GLS measured from CMR (17).

The decision about where to make the cutoff to recommend further investigation depends on whether sensitivity or specificity is the predominant clinical priority (Table 3). If the cutoff was set at a score of at least 2 (100% sensitivity and 42% specificity), it would ensure that the vast majority of silent MI was detected with only 2.5 patients needing CMR to identify one patient with silent MI. Alternatively if the cutoff was higher with a score of at least 3 (82% sensitivity and 72% specificity), approximately one in six patients with silent MI would be missed, but only 1.5 patients would need to be screened to detect one patient with silent MI. Either of these two scenarios are vastly superior to our current screening test using Q waves that only had 24% sensitivity in our asymptomatic cohort and 28% sensitivity in a cohort of patients with clinically recognized non-ST elevation MI (7).

All of the measured components within the score, impaired GLS (18), elevated NT-proBNP (19), and E/A ratio (20) have been associated with adverse outcomes in patients with type 2 diabetes without prior history of MI. It is likely that a proportion of the mortality reported in these patients is due to silent MI. It has also been shown that larger infarcts have greater impairment of GLS (9), higher NT-proBNP (11), and altered mitral inflow (21). Therefore all of the measured parameters have biological validity and prognostic significance that supports their inclusion in a risk score.

The imaging parameters associated with mass of silent MI were different from those included in the silent MI risk score and included LV ejection fraction, E/E′, and hs-cTnT. These parameters are all recognized to correlate with extent of infarction and prognosis after symptomatic MI (8, 10, 22); however, we have demonstrated that they were insensitive for the detection of silent MI in type 2 diabetes and of limited value in this setting.

It was an unexpected finding that conventional risk factors including fasting cholesterol, 24-hour BP, smoking, and even previous glycemic control had no association with the likelihood of silent MI in our population, although this may reflect a relatively small sample size and appropriate use of primary prevention medication. However, it is unclear whether the pathological processes that lead to silent MI are identical to acute MI. The lack of association with conventional risk factors suggests that further research is needed to identify alternative risk factors specifically for silent MI.

To our knowledge this is the first study to assess silent MI by LGE CMR in a truly asymptomatic diabetic population. Previous studies have demonstrated that in patients with diabetes, silent MI detected on CMR is associated with increased mortality and adverse cardiovascular events (3, 4). Kwong et al reported an incidence of silent MI of 28% in symptomatic patients with diabetes undergoing clinical CMR (3). Schelbert et al reported a prevalence of 21% of silent MI of diabetic patients enrolled in the ICELAND MI study who underwent CMR between 2004 and 2007 (4). However, patients in both studies were not necessarily asymptomatic and in ICELAND MI, 28% of those with silent MI had prior coronary revascularization. The rate of infarction was similar between our study and the work of Schelbert et al, despite patients in our study being younger, lower risk, and asymptomatic.

Despite recommendations of aggressive risk factor modification in type 2 diabetes uptake remains variable (23) (with only 18% taking aspirin at time of recruitment to this study). Recognition of silent MI in these patients should prompt aggressive risk factor modification, which may improve long-term clinical outcome. Furthermore, the silent MI screening components that we have identified may help in future clinical studies by identifying those most likely to have silent MI who could be targeted with lifestyle, pharmacological, or interventional management.

### Limitations

There several limitations to this work that should be acknowledged. First, we have excluded certain higher risk patients—for example, those on insulin or angiotensin-converting enzyme inhibitors—therefore, general applicability of the findings is uncertain. The silent MI risk model we propose would need to be validated in more varied populations to broaden its clinical use. Second, we have not performed coronary angiography to confirm that silent MI was caused by coronary disease; however, in an asymptomatic cohort, undertaking an invasive procedure would not be appropriate. Third, we have recruited a relatively low proportion of women; however, previous data suggest that the rate of silent MI tends to be equal or lower in women (3, 4). Finally, the cutoff points that we have used are based on Youden index, which assigns equal importance to sensitivity and specificity. Depending on which of these is more important in clinical practice, the thresholds would need to be altered accordingly. We have also assigned an equal score to each of the components, which may oversimplify the complex nature of the disease process.

## Conclusions

The rate of silent MI in this low-risk asymptomatic cohort of patients with type 2 diabetes was higher than expected. Several simple clinical parameters including age, E/A ratio, GLS, and NT-proBNP were associated with silent MI. By combining them, we were able to define a novel screening tool with superior diagnostic accuracy than current ECG criteria for the detection of silent MI, which can be used both clinically and for interventional studies.
